# Evolving competencies to align electronic medical records – a dynamic resource-based perspective on hospitals' co-evolutionary information systems alignment capability

**DOI:** 10.1108/JHOM-10-2021-0379

**Published:** 2022-03-30

**Authors:** Pien Walraven, Rogier van de Wetering, Remko Helms, Marjolein Caniëls, Johan Versendaal

**Affiliations:** Open University of the Netherlands , Heerlen, The Netherlands; HU University of Applied Sciences Utrecht , Utrecht, The Netherlands

**Keywords:** Electronic medical records, Co-evolutionary information systems alignment, Capability evolution, Dynamic resource-based perspective, Business-IT alignment

## Abstract

**Purpose:**

Advanced Electronic Medical Records (EMR) provide many potential benefits to hospitals. However, because of their broad scope, many stakeholders deal with the EMR and a continuous effort has to be made to keep up with internal and external change. Therefore, hospitals need to deliberately shape their organizational competencies considering the pursuit of alignment, i.e. making sure that the EMR remains optimally aligned with strategies, goals and needs of the hospital and its stakeholders. This paper aims to investigate the evolutionary paths of these alignment competencies and their drivers, from a theoretical perspective of co-evolutionary information systems alignment (COISA).

**Design/methodology/approach:**

This paper reports on a longitudinal multiple case study of three Dutch hospitals which each recently implemented an advanced EMR system. The authors conducted 35 in-depth interviews in 2 phases (before and after go-live of the EMR), and studied documentation related to the EMR implementations.

**Findings:**

The findings show that each hospital's COISA capability shows a different evolutionary path. However, two of the three case hospitals ended up coordinating part of their COISA capability to an ecosystem level, i.e. they incorporated other hospitals using the same EMR system to coordinate their alignment efforts, either from an operational perspective, or in terms of orchestration and strategy. The found evolutionary paths' key drivers include “stakeholder initiative”, “accumulating experience”, “driving events” and “emerging issues”.

**Originality/value:**

The findings help healthcare practitioners to deliberately shape their organization's COISA capability in pursuit of EMR alignment. Furthermore, the authors add to the knowledge base on co-evolutionary approaches to alignment through the longitudinal approach.

## 1. Introduction

Electronic Medical Records (EMR) have become an increasingly important resource for modern healthcare in Western countries, with many contemporary hospitals rapidly implementing advanced EMR. Traditionally, these EMR involve electronic repositories of patients' medical histories ( Kohli and Tan, 2016 ). However, advanced EMR provide many additional functionalities and advantages such as hospital-wide integrated information, medical decision-support and direct patient access via patient portals ( Carvalho *et al.* , 2019 ). As a consequence, EMR become interdependent with an increasing amount of processes. Through these developments, many stakeholders deal with EMR, all having their own views on how to apply the EMR appropriately ( Davidson *et al.* , 2018 ). Given these different and sometimes contradicting interests, it is challenging to develop and maintain the EMR such that it optimally aligns with the strategies, goals and needs of the hospital and its stakeholders. Coping with this complexity requires hospitals to actively shape their capabilities to reach and maintain a certain degree of alignment of their EMR ( Walraven *et al.* , 2020 ). In doing so, potential benefits of the EMR such as cost savings, improved patient experience and better decision-making can be leveraged.

The specific conditions characterizing EMR in hospitals are exemplary of complex conditions where co-evolutionary approaches to alignment capabilities are argued to be useful ( Amarilli *et al.* , 2017 ; Benbya and McKelvey, 2006 ). While some traditional approaches to alignment view the concept as an end-state ( Chan and Reich, 2007 ), this relatively new approach involves multi-level effects, multi-directional causalities, non-linearity and feedback loops, with a focus on the *“[…] series of co-evolutionary moves that makes IS aligned over time”* ( Benbya and McKelvey, 2006 , p. 288). Furthermore, more recent conceptualizations of co-evolutionary information systems alignment (COISA) expand this dynamic approach to alignment by applying a stakeholder interaction perspective in pursuit of alignment ( Allen and Varga, 2006 ). Thereby, these works explicitly address earlier criticisms on traditional alignment conceptualizations emerging as early as 1997 when Ciborra (1997 , p. 79) underlined the blurring boundaries between business and information technology (IT) and the importance of working towards “ *An enlarged notion of alignment within a hybrid network of semi-autonomous actors.”*

Given the complexity faced by contemporary hospitals in search of better EMR alignment, researchers have applied this co-evolutionary approach to alignment to EMR before ( Walraven *et al.* , 2020 ). This particular work illustrates that COISA could indeed be a fruitful way to understand the capabilities that hospitals need to effectively execute and maintain an inclusive, two-way dialogue in pursuit of better aligned EMR ( Walraven *et al.* , 2020 ). Thus far, these endeavors focus on the implementation phase of the EMR. However, given the many different stakeholders and the continuously changing external conditions, it is unlikely that the EMR is and will remain optimally aligned with the needs of the hospital and its stakeholders at the point of go-live. Therefore, hospitals will also need to develop and maintain their COISA capability after go-live to continuously align the EMR and optimally leverage potential benefits. Still, existing empirical works on EMR-related COISA capabilities focus on the EMR implementation phase only ( Walraven *et al.* , 2020 ). Therefore, it remains unclear what drives the evolutionary paths of the COISA capability, and thus hospitals have little guidance on how to best shape EMR alignment in the long run. We contribute to closing this knowledge gap by giving further insight into how the evolutionary path of the COISA capability develops before and after EMR go-live in hospitals and in the drivers behind these evolutionary paths. Hospitals may use these insights to better shape their COISA capability to maintain an adequate degree of alignment. Our research question is as follows:

RQ. How does the EMR-related COISA capability evolve in hospitals, and what are key drivers for how this capability evolves?

To address this question, we did a longitudinal multiple case study, where we examined the EMR-related COISA capability in three hospitals using in-depth retrospective interviews regarding two different phases, i.e. before EMR go-live and after EMR go-live. A longitudinal approach is appropriate because our goal is to look at the COISA capability's evolution over time, which fits the logic of selecting a longitudinal approach ( Yin, 2018 ).

## 2. Theoretical framework

**Figure 1:**
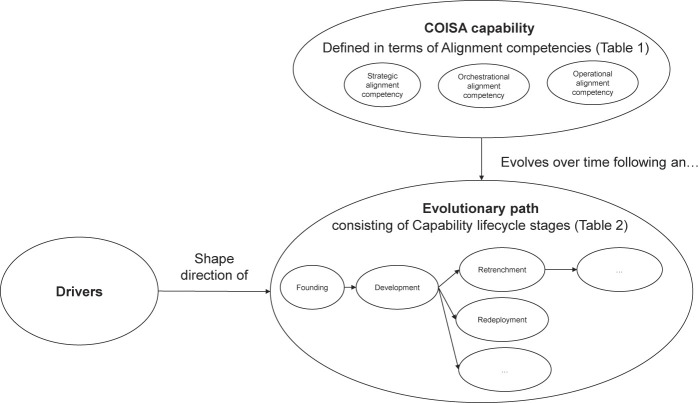
Conceptual model of our current study, researching evolutionary paths of the COISA capability and their drivers

This chapter outlines our research's theoretical foundation, as illustrated in Figure 1 . We will elaborate on each of the concepts of this model, including COISA as an organizational capability consisting of alignment competencies; describing capability evolution in terms of capability lifecycle stages following the dynamic resource-based perspective; and finally describing potential informative works on drivers of the COISA capability evolution in an EMR context. In this current study, we approach the COISA capability as alignment competencies in continuous pursuit of EMR alignment. Specifically, EMR alignment entails a common (i.e. held across EMR stakeholders) interpretation and implementation of what it means to apply the EMR in an appropriate and timely way, in harmony with strategies, goals and needs of the hospital and its stakeholders ( Luftman and Brier, 1999 ; Luftman and Kempaiah, 2007 ; Walraven *et al.* , 2020 ). This definition resonates with the concept of social alignment, i.e. *“[…] when groups share understanding and commitment towards an outcome, and the means of achieving that outcome”* ( Gilchrist *et al.* , 2018 , p. 1), with the extension that EMR alignment not only incorporates the common interpretation across stakeholders, but also its practical implementation in terms of the configuration of the EMR and other hospital resources. Furthermore, because of the continuous change present in and around the hospital, EMR alignment is not an end-state, but a moving target, hence the need for a long-term alignment capability to pursue it ( Baker *et al.* , 2011 ; Vessey and Ward, 2013 ).

### 2.1 Co-evolutionary information systems alignment

COISA is a stream of alignment research suitable for complex conditions ( Allen and Varga, 2006 ; Amarilli *et al.* , 2016 ; Benbya and McKelvey, 2006 ; Walraven *et al.* , 2020 ). This school of thought focuses on the social actors comprising organizations and their co-evolutionary interactions in pursuit of alignment. These interactions prevail within and between individual, operational and strategic levels of the organization ( Benbya and McKelvey, 2006 ), which consists of several alignment processes, including strategy formulation ( Kahre *et al.* , 2017 ; Liang *et al.* , 2017 ; Sabherwal and Chan, 2001 ), strategy implementation ( Busquets, 2015 ; Grisot *et al.* , 2014 ; Liang *et al.* , 2017 ; Montealegre *et al.* , 2014 ), enterprise architecture management ( Schilling *et al.* , 2017 ; Vessey and Ward, 2013 ; Weeger and Ulrich, 2016 ), IT implementation ( Lyytinen and Newman, 2008 ; McLeod and Doolin, 2012 ; Wagner *et al.* , 2010 ) and IT usage ( Allen *et al.* , 2013 ; Burton-Jones and Gallivan, 2007 ; Goh *et al.* , 2011 ). The theoretical foundations of COISA are in line with a broader research area that positions alignment as a continuous process, emerging from networks of actors in the flow of organizational practice ( Ciborra, 1997 ).

In this current study, we conceptualize COISA as an organizational capability, consisting of three continuously exercised alignment competencies characterized by co-evolutionary interactions between heterogeneous information systems (IS) stakeholders, in pursuit of a common interpretation and implementation of what it means to apply IT in an appropriate and timely way ( Walraven *et al.* , 2021 ). In doing so, we distinguish three alignment competencies that are based directly on the abovementioned alignment processes as synthesized from the literature by Walraven *et al.* (2018) and the different levels of alignment as outlined by Benbya and McKelvey (2006) . These competencies include the: strategic alignment competency, orchestrational alignment competency, and operational alignment competency. Table 1 summarizes our definitions of each of these alignment competencies, as defined by Walraven *et al.* (2021) and based on leading articles using dynamic perspectives on alignment (e.g. Baker *et al.* (2011) , Liang *et al.* (2017) , Vessey and Ward (2013) , Vidgen and Wang (2009) and Weeger and Ulrich (2016) ). This dynamic perspective particularly fits our research context of EMR as it is specifically hypothesized to be better able to deal with internal and external complexities ( Merali *et al.* , 2012 ; Merali and McKelvey, 2006 ):

### 2.2 COISA as an organizational capability

In line with the conceptualization as explained by Walraven *et al.* (2021) , we view COISA as a whole as an organizational capability, with the three above described alignment competencies as its foundation. This stance builds upon several existing works in the field of organizational capabilities, including Peppard and Ward (2004) , who define organizational capabilities as *“[…] the strategic application of competencies […],* i.e. *their use and deployment to accomplish given organizational goals”* . In line with this definition, the COISA capability considers the use and deployment of the strategic, orchestrational and operational alignment competencies. Their combination, in particular, makes for a *strategic* application. Crick and Chew (2020 , p. 12) argue that business processes are *“[…] the basis for an organization's capabilities and how they “earn their living”.* This also resonates with COISA: Namely, as shown in earlier works on COISA, alignment processes form the micro-foundations of the strategic, orchestrational and operational alignment competencies ( Walraven *et al.* , 2020 , 2021 ), which in turn comprise the COISA capability.

Helfat and Peteraf (2003 , p. 999) define an organizational capability as *“[…] the ability of an organization to perform a coordinated set of tasks, utilizing organizational resources, for the purpose of achieving a particular end result”* . COISA fits well into this framework since a crucial part of COISA relies on coordinating tasks not only within but particularly between the different alignment competences. This coordination takes place *through* the social interactions between stakeholders ( Walraven *et al.* , 2020 ). Moreover, this perspective is resonant with the insight that the micro-foundations of capabilities consist of individuals interacting within and between organizational and managerial processes ( Harris *et al.* , 2013 ). Thus, COISA as an organizational capability considers the combination and successful application of the three different alignment competencies to continuously align IT.

However, viewing alignment as a capability is not novel. For example, earlier research has suggested that strategic alignment maybe conceptualized as a collection of complementary capabilities, including (1) dynamic capabilities, (2) IT flexibility and (3) absorptive capacity ( van de Wetering and Mikalef, 2017 ). Still, as this study also points out, this particular conceptualization remains quite general, and *“Future work could address how managers should deploy improvement projects done simultaneously and hence by an integrated alignment perspective”* ( van de Wetering and Mikalef, 2017 , p. 10). Other researchers that address the alignment challenge using a capability perspective include Baker *et al.* (2011) . These authors conceptualize strategic alignment as *“an enduring competency that is a source of competitive advantage”* . However, in their study, they only focus on aligning IT strategy with business strategy, and thus do not consider individual and operational levels of alignment. The only study that takes an explicit co-evolutionary stance on the conceptualization of alignment as an organizational capability is the one by Walraven *et al.* (2021) , which is why we use that conceptualization as the theoretical foundation of the current study.

### 2.3 COISA as an evolving capability

Given our conceptualization of COISA as an organizational capability that needs continuous attention and effort, the question arises whether, how and in what circumstances the COISA capability evolves over time. Several researchers have looked into the theoretical foundations of the evolution of capabilities. Helfat and Peteraf (2003) argue that dynamic and organizational capabilities both can be described to have life cycles, consisting of three generic stages, i.e. (1) Founding stage, (2) Development stage and (3) the Maturity stage. A given capability can “branch” into different directions during the development or maturity stages. Table 2 summarizes the characteristics of each of these capability lifecycle stages. For this current study, we use the capability lifecycle stages by Helfat and Peteraf (2003) as a starting point.

### 2.4 Key drivers of COISA capability evolution in an EMR context

Several studies have written about drivers of evolutionary paths of organizational capabilities. Helfat and Peteraf (2003) , whose capability lifecycle stages we use as a conceptual lens, argue that branching of a capability into another stage is triggered by selection events. These consist of threats to the capability or opportunities for the capability to grow. Furthermore, Zollo and Winter (2002) take a knowledge-based view and argue that the micro-foundations of the evolution of dynamic capabilities lie in learning mechanisms, including experience accumulation, knowledge articulation and knowledge codification. Moreover, resource orchestration theory suggests that managerial actions can be seen as potential drivers of capability evolution ( Sirmon *et al.* , 2011 ). This particular theory combines insights from resource management and asset orchestration to look deeper into the role of managers' actions in the effective structuring, bundling and leveraging of firm resources in pursuit of competitive advantage. In other words, from this perspective, the formation and evolution of organizational capabilities is explicitly seen as a managerial responsibility, and managers' actions are hypothesized to shape these orchestration efforts. This premise has been empirically demonstrated and holds for executive and operational and middle managers ( Chadwick *et al.* , 2015 ; Sirmon *et al.* , 2011 ). Pelletier *et al.* (2021) looked into social IT alignment (SITA) through an asset orchestration lense and find that SITA is facilitated through the allocation, structuring and coordination of IT resources and that “ *[…] proper management of the SITA process is founded on the exchange and sharing of IT competencies and knowledge”* ( Pelletier *et al.* , 2021 , p. 3).

In terms of specifically healthcare IT- or EMR-related capability drivers, several works give insight into the potential drivers of related capability evolution. Sha *et al.* (2011) find in their case study on the implementation success of healthcare information systems that normative pressure, top management support, domain knowledge sharing and having a culture of innovation drives the development of the alignment capability. Palvia *et al.* (2015 , p. 711) describe that the EMR vendor often has a large influence in the development of capabilities during EMR implementations: *“[…] the* [EMR] *vendor's active participation in the EHR implementation is necessary due to project management and change management expertise that the vendor possesses and that maybe missing or insufficient in the healthcare organization”* . Additionally, Walraven *et al.* (2020) describe facilitators of efficacious co-evolutionary stakeholder interactions toward alignment during EMR implementations based on the literature on effective alignment (e.g. Amarilli *et al.* , 2017 ; Zhang *et al.* , 2019 ) and efficacious dynamics in complex organizations (e.g. Burt, 1992 ; Grant, 2003 ; McKelvey, 2001 ) and applied in an empirical EMR setting. These facilitators may be seen as drivers of capability founding and development since they contribute to the growth of the COISA capability. However, they do not explicitly give insights into the drivers of the other capability lifecycle stages, except that maybe the absence of one or more of these facilitators could lead to the capability's retrenchment or retirement. The facilitators described by Walraven *et al.* (2020) include alignment motivation, considering *“[…] facilitators motivating IS stakeholders to engage in co-evolutionary interactions in a specific alignment process”* ( Walraven *et al.* , 2020 , p. 9); Stakeholder involvement, or *“[…] facilitators related to the selection of actors to be involved in COISA processes”* ( Walraven *et al.* , 2020 , p. 9) *;* Interconnections, or “ *[…] the means that IS stakeholders have to engage in co-evolutionary alignment interactions”* ( Walraven *et al.* , 2020 , p. 11), and finally alignment decisions, considering *“[…] specific decisions that are taken in the alignment processes themselves and that may, in turn, benefit following COISA interactions in those same processes”* ( Walraven *et al.* , 2020 , p. 11). These alignment decisions include having common guidelines, putting in place central coordination of the COISA capability, allowing emergent decision-making, and having the adequate technical infrastructure in place.

## 3. Methodology

We conducted a longitudinal multiple case study through retrospective interviews in two phases. Our multiple case approach improves generalizability since it enables us to compare the evolution of the COISA capability across multiple hospitals ( Yin, 2018 ). We used a longitudinal approach because we are interested in the evolution of the COISA capability over time. The first interview phase focused on the EMR implementation and its preparation and was carried out in the months right after the go-live of the EMR system in each hospital. Herein, we studied whether and how a COISA capability was founded to align the EMR prior to or during the implementation, and how the capability evolved in each of the studied hospitals during this period. The second interview phase was done during the six to twelve months after go-live of the EMR, to study and reflect on how the COISA capabilities further evolved after the initial go-live. The two-phased approach to longitudinal research is comparable to the longitudinal study method on stakeholder roles and perceptions in health information systems by Pouloudi *et al.* (2016) .

Furthermore, our approach aligns with the before-and-after logic for longitudinal research ( Yin, 2018 ), where data collection is done in two phases, i.e. before and after a critical event. In our current study, this critical event entails the go-live of the EMR. We will now elaborate on each of the three case studies.

### 3.1 Case studies

We selected three Dutch hospitals based on two criteria: First, they all implemented a new, advanced EMR in the past few years, and second, that they are academic or top clinical hospitals. The latter criterion ensured the presence of complexity that the COISA capability is hypothesized to address. All hospitals implemented a vendor-built system: Hospital A and Hospital C opted for a vendor (Vendor 1). This vendor originates from the United States and implemented their EMR solution in countries across the globe including the United Stated, Canada, England and the Netherlands. They offer an EMR solution that is standardized to a certain degree but still has many configuration possibilities for individual hospitals. Hospital B implemented a standardized EMR system from a different vendor (Vendor 2), which is also standardized and to some degree configurable. However, it is much less flexible than the system from Vendor 1. Vendor 2 is the Dutch market leader, having implemented their EMR system at 70% of all Dutch hospitals ( van Eekeren *et al.* , 2021 ). Hospital A and Hospital B both went through a merger simultaneous to the EMR implementation. These mergers were for both of these case hospitals, a main reason to implement a new EMR. Hospital C did not go through a merger, but its existing EMR was soon reaching its end-of-life.

Furthermore, the hospitals all carried out some preparations before the actual implementation program of the EMR, however, the scope and time spent on that pre-implementation phase differed for each case: Hospital A had the most extensive pre-implementation phase, considering an entire program focusing on process- and IT harmonization in preparation of the upcoming merger. Hospital B was also going through a merger and aimed to harmonize processes as much as possible before the EMR implementation. However, they did not set up a separate program to this end. Instead, they gave department heads the responsibility to pursue this before the implementation phase would start. Hospital C had a minimal pre-implementation phase, because of time limits and presumably because they did not face a simultaneous merger. The time limits were caused by a previously failed implementation of a different EMR, leaving the hospital very limited time before the end-of-life of their previous EMR. Furthermore, unlike Hospitals A and B, Hospital C opted for a two-staged implementation of the EMR of Vendor 1, to make sure that the most crucial EMR parts could go live in time. Specifically, in the first implementation stage, the hospital implemented the essential EMR modules to enable the different departments to administer and exchange patient information and support essential healthcare processes. In the second implementation stage, the hospital worked on optimizing these functionalities and added additional functionalities such as mobile apps for doctors and nurses, information exchange possibilities with general practitioners and integrations with medical devices. Table 3 summarizes relevant case information.

### 3.2 Data collection approach

We conducted in-depth interviews with key stakeholders in two phases. In doing so, we aimed for an optimal stakeholder representation (IT; external; management and medical), as recommended by Pouloudi *et al.* (2016) . Furthermore, we selected respondents with knowledge of the whole implementation program and the EMR, i.e. respondents who are strategically responsible for the EMR and its alignment and overview all relevant aspects. These stakeholders are most likely to have knowledge on the formation and evolution of formal governance structures in relation to EMR alignment. Moreover, we aimed to interview people whose primary role in relation to the EMR was to represent their constituencies during and after the implementation. For example, in hospital C, the digital doctors represented all doctors in the hospital in the decision-making around the EMR. The rationale behind this particular criterion is to optimize the stakeholder representation in our data and to get a better idea of informal influences on decisions related to EMR alignment on lower hierarchical levels. We could not cover the patient perspective, because we could not identify a representative of this group meeting this latter criterion. We could not include the vendor's perspective for hospitals A and C because this vendor was unwilling to participate in our study. To identify suitable respondents for the second phase, we used the same selection criteria and first contacted our first-phase respondents. Then, depending on whether they were still actively involved with the EMR, we interviewed them second time or asked them to refer us to suitable respondents. Our first data collection phase (phase I) took place between September 2018 and November 2018. Our second data collection phase (phase II) took place between March 2019 and June 2019. We finally interviewed at least five respondents per hospital per phase, amounting to 35 interviews in total, as summarized in Table 4 .

Our questions focused on the respondent's experience with decision-making and stakeholder involvement on different levels during and after EMR implementation. We aimed to cover different levels by asking about the EMR implementation operationally, but also about the role of strategic and architectural practices (if present); in doing so, we made sure the interview's themes were congruent with the three alignment competencies and their microfoundations (i.e. alignment processes). Furthermore, we asked each respondent to elaborate on their role in relation to the EMR during implementation and/or after go-live. Specifically, we focused on how their role related to any official governance structures and how they played a role in any informal decision-making structures. We also asked about how these formal and informal decision-making structures and EMR alignment related decisions evolved during the implementation and after, and how the involvement and stance of the different stakeholder groups evolved over time and why. This latter effort ensured that we could analyze and compare data on how the alignment competencies and their microfoundations evolved over time. To enable data triangulation, we also collected documentation related to the EMR, including project plans, strategic guidelines and decision-making structures.

### 3.3 Data analysis approach

All interviews were recorded, transcribed and coded. The coding process was informed by the recommendations by Saldaña (2015) and involved a three-step approach: First, passages that indicated a particular capability life cycle stage were coded using a deductive approach, based on the work by Helfat and Peteraf (2003) . Then, each of the coded passages was labeled in a second round as either considering the operational, the orchestrational or the strategic alignment competency. Lastly, we went through the entire dataset once more to identify and categorize the drivers of the COISA capability's evolutionary paths. To improve reliability, we pursued inter-coder agreement levels as follows: The resulting analysis and corresponding coding were independently reviewed by two other researchers, who coded the interview passages in terms of alignment competency and capability lifecycle stage. When disagreements arose, we had substantial discussions to come to a final analysis.

## 4. Results

Our results show that (parts of) the COISA capability of all three hospitals went through the founding-, development-, retrenchment- and renewal stages. Furthermore, in Hospital B, parts of the COISA capability went into the redeployment stage. Finally, our data analysis revealed a stage that was not included in the original model by Helfat and Peteraf (2003) , which we named the “coordination” stage. We characterize this “coordination” stage as follows: a capability founded *within* organizational boundaries is brought to a higher *network- or ecosystem-level* by formally incorporating other organizations in the capability. In two of the three case study hospitals, part of the COISA capability evolved in this direction after go-live.

**Figure 2:**
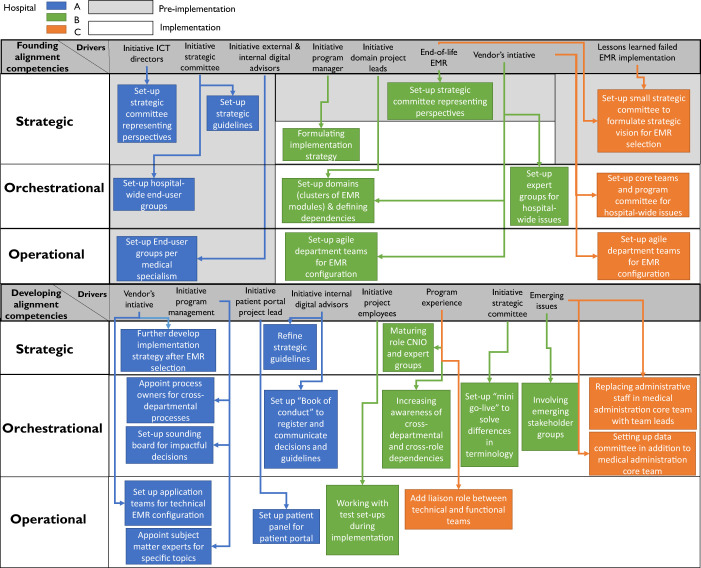
Results of data collection Phase I

**Figure 3:**
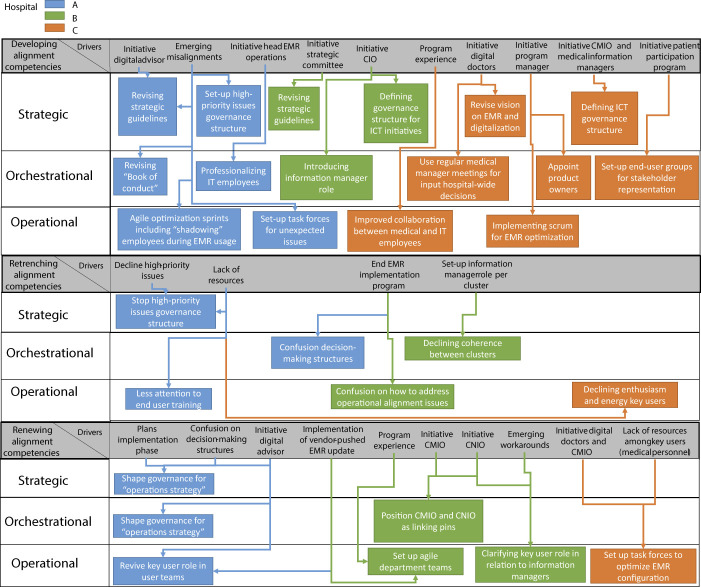
Results of data collection phase II (Results manifesting in all three cases)

**Figure 4:**
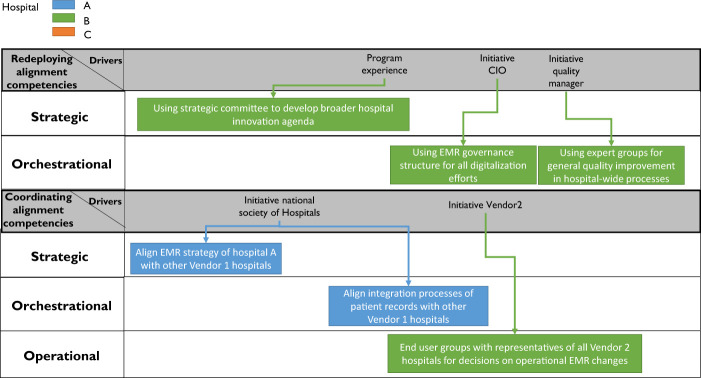
Results of data collection phase II (Results manifesting in one or two cases)

In the remainder of this chapter, we will elaborate on the evolutionary paths of the COISA capability and their drivers, highlighting similarities, differences and possible explanations for our findings. In doing so, we will first go into the evolutionary paths of the COISA capability during the (pre)-implementation phase of the EMR, i.e. the results from our first data collection phase (see Figure 2 ). Then, we will elaborate on our findings from our second data collection phase, considering the six to twelve months after go-live of the EMR in our three case hospitals (see Figures 3 and 4 ). We will finally focus on the particular drivers of the COISA capability evolution, both during (pre-)implementation and after go-live.

### 4.1 Data collection phase I: before go-live

Figure 2 summarizes all capability lifecycle stages manifesting during (pre-)implementation of the EMR, before go-live, i.e. the founding stage and the development stage. Each grey row in this figure represents a particular capability lifecycle stage and its drivers that we identified in our case studies. Furthermore, for each of these stages, the specific actions forming the practical manifestations of that particular stage are categorized in three white rows, one per alignment competency. For the development stage, we differentiated between actions performed during the pre-implementation phase and during the implementation phase of the EMR. Lastly, all actions are color-coded so that it is clear which action manifested in which case (Hospital A is blue, Hospital B is green and Hospital C is orange).

#### 4.1.1 Implementation: founding and development of the COISA capability

While hospital A had already founded all alignment competencies during pre-implementation, hospitals B and C founded their operational and orchestrational alignment competencies during the implementation, shaped by the EMR vendors' implementation strategy. Following, all three hospitals' COISA capabilities evolved into the development lifecycle stage during the EMR implementation.

In hospital A, the initial implementation strategy set up during pre-implementation was incrementally updated based on input from the vendor, as pointed out by several interviewees (Project lead A, hospital A; Project lead D, hospital A). Based on these incremental updates, hospital A's strategic alignment competency developed into hybrid between the vendor's standardized implementation strategy and hospital A's own experience during the pre-implementation phase. For example, hospital A kept their process-focused hospital-wide and specialism-specific end-user groups, even though those were not usually part of the governance structure prescribed by the vendor: *“You have to be well-prepared because this vendor works mainly around applications, while we had deliberately set up our end-user teams around processes. In the beginning, this really was a struggle to keep it that way. But we believed in what we were doing: we felt like we knew why we did it that way. But we had to justify ourselves”* (Project lead D, hospital A). Furthermore, several internal actors in hospital A initiated some additional developments of the COISA capability. For example, in terms of hospital A's orchestrational alignment competency, process owners were appointed for cross-departmental processes such as outpatient clinic logistics (ICT manager, hospital A). Moreover, the “book of conduct” was set up as a development of the hospital's orchestrational alignment competency: *“In the book of conduct, all hospital-wide decisions were documented. So, for example, who is authorized to order medicine, and what kind of medicine?”* (Project lead B, hospital A).

In hospitals B and C, the COISA capability's development was influenced by their acquired experience during the implementation. For example, in hospital B, experience during the program caused the orchestrational alignment competency to evolve: *“While making configurational decisions while working together, people really saw through the entire process of a patient coming in at first aid, through the entire chain of departments including the operating room, and there really was some kind of realization. […] We really made some big steps there, also in terms of cross-departmental process harmonization.”* (ICT architect, hospital B). A comparable mechanism emerged in hospital C: *“Most hospitals do not have much experience with these types of implementations. So in the beginning, the vendor is very much in the lead in the way operational decisions are being made. […] And as the implementation moves forward, you become more of a partner and the vendor gradually moves to the background and you start to shape things your own way.”* (Program manager 1, hospital C).

### 4.2 Data collection Phase II: after go-live

In our second data collection phase, we identified three lifecycle stages in all of our case hospitals (i.e. development, retrenchment and renewal) and two in only one or two of our case hospitals (i.e. redeployment, and a new lifecycle stage called “orchestration”).

#### 4.2.1 Development, retrenchment and renewal of the COISA capability

After go-live of the EMR, the COISA capability of all case hospitals showed evolutionary paths of development, retrenchment and renewal (see Figure 3 ).

In hospital A, the development of the capability consisted of two important types of actions. Firstly, these involve actions to address urgent issues emerging after go-live of the system. For example, in terms of its operational alignment competency, hospital A set up so-called task forces to solve these most urgent issues (project lead D; digital nurse; head EMR operations, hospital A). Secondly, these involve actions focused on improving existing structures to form a solid foundation for an effective COISA capability during EMR operations. For example, strategic guidelines were revised to further develop the strategic alignment competency, and agile optimization sprints including personal training based on “shadowing” were initiated as part of the operational alignment competency: *“All outpatient clinics get an agile sprint during three weeks, and then all doctors in those clinics are being “shadowed” […] to be able to analyze: how can this person make better use of the EMR? And during those same three weeks we look at, what are the department's wishes in terms of optimization and development of the EMR?”* (project lead training, hospital A).

At some point after go-live, hospital A's COISA capability started to show signs of retrenchment, for example because there was less attention to end user training (digital doctor; project lead training, hospital A) and because there was confusion about how decision-making structures were supposed to work after the end of the implementation program (digital doctor, hospital A). The capability was then renewed in several ways, for example, using the momentum of a vendor-pushed EMR update to revive the key user role (digital nurse, hospital A).

In Hospital B, the development of the COISA capability, like in Hospital A, also involved solving urgent issues on the one hand and working towards a more mature COISA capability during EMR operations on the other hand. For example, this hospital also set up agile teams as part of their operational alignment competency, addressing both urgent issues and less urgent issues focused more on optimization (information manager; ICT architect; hospital B). Comparable to Hospital A, hospital B's COISA capability also evolved towards retrenchment after go-live, for example because of the confusion that emerged after the end of the implementation program on how to address operational issues (ICT architect; CNIO, hospital B). The operational alignment competency was in turn renewed by setting up agile department teams to optimize EMR configurations (Head EMR operations, ICT architect, hospital B).

In hospital C, the COISA capability's development was visible for example in the revision of the hospital's vision on the EMR (digital doctor 1; digital doctor 2; hospital C). Moreover, there was a parallel effort by the CMIO and several information managers to set up a governance structure for EMR alignment during operations: *“EMR operations are now situated in the implementation program and not in the regular organizational structure of the hospital. […] What we are working on right now is the transition from the program to the regular organizational structure, towards the department of information management. Together with those information managers, I will be responsible for all healthcare related information technology, including the EMR”* (CMIO, hospital C). Although not as clearly visible as in Hospitals A and B, Hospital C also showed some signs of COISA capability rentrenchment because of the declining enthusiasm of key users: *“[…] enthusiasm is declining because many healthcare employees are back to their own work”* (digital doctor, hospital C). This particular hospital addressed this declinement by renewing the operational alignment competency, i.e. by setting up task forces: “ *the optimization teams visit all outpatient clinics in the hospitals to see what issues they face in the EMR, to be able to quickly fix emerging issues and optimize the EMR.”* (digital doctor, hospital C).

#### 4.2.2 Redeployment and coordination of the COISA capability

We identified the redeployment lifecycle stage in one of our case studies (hospital B) and the new “coordination” stage in two of our case studies: hospitals B and C (see Figure 4 ).

In hospital B, we found that after go-live, parts of the COISA capability were *redeployed* to IT-related innovations other than the EMR. For example, departments were encouraged to “pitch” innovative IT solutions (not necessarily within the EMR), supported by the newly founded role of information manager (present at each group). (ICT Architect; Information manager; CNIO, hospital B). The digital strategic committee that was initially founded as part of the EMR program was redeployed in this context because it reviewed and prioritized these pitches. Moreover, they developed a digital strategy broader than just the EMR after go-live (CNIO, CIO, Hospital B).

In Hospitals A and B, we identified a new lifecycle stage of the COISA capability relative to the predefined lifecycle stages based on Helfat and Peteraf (2003) . We have named this particular stage the “coordination” stage. This capability lifecycle implies that a capability founded on an organizational level is extended to a broader ecosystem. This extension is done by formally incorporating other organizations in the capability to collaboratively coordinate alignment efforts, hence the name “coordination”. In our empirical findings, these other organizations entailed other hospitals working with the same EMR. However, the particular focus of coordination differed depending on the vendor's strategy. The COISA capability of Hospital B, working with vendor 2's highly standardized solution, evolved toward the coordination stage in the operational alignment competency. Namely, to ensure that the highly standardized solution fits the hospitals that have implemented this solution as much as possible, vendor 2 has set up their end-user groups consisting of representatives of the different hospitals using vendor 2's EMR (CNIO; Vendor representative, hospital B): “ *For this standardized solution, we set up end-user groups. This is on the level of medical specialists, and they periodically meet. For example, we have an end-user group cardiology, consisting of cardiologists of all the hospitals working with our standardized solution. […] And the customers are in charge, so these groups' chairs are also representatives from one of the hospitals. We* [Vendor 2] *facilitate these meetings […] The benefit is that when consensus on a specific topic emerges among the different hospitals, we can adapt our standardized solution to their needs, and everyone is happy.”* (Vendor representative, hospital B). In short, these end-user groups make decisions on specialism-specific issues that occur on an operational level, and can thus be considered to be a vendor-facilitated, cross-hospital execution of the operational alignment capability.

The COISA capability of Hospital A, working with vendor 1 and its slightly less standardized solution, evolved toward the coordination stage in the orchestrational and strategic alignment competencies. The head of EMR operations elaborated: *“There is the directors' meeting among all hospitals working with the EMR solution of this vendor […] So you have all the national hospitals working with Vendor 1 there, and Vendor 1 is represented as well. The idea is to exchange knowledge, to set up contacts and to have a strong position in certain themes, priorities or developments in relation to vendor 1.”* (Manager healthcare, hospital A). Coordination of the orchestrational alignment competency was done across hospitals working with vendor 1's EMR, for the theme of data integration (project lead A, hospital A)

### 4.3 Key drivers of the COISA capability evolution

Based on our literature review, we identified several potential drivers of capability evolution including (1) Selection events ( Helfat and Peteraf, 2003 ); (2) Managers' motivation ( Chadwick *et al.* , 2015 ; Sha *et al.* , 2011 ; Sirmon *et al.* , 2011 ; Walraven *et al.* , 2020 )); (3) Accumulating experience ( Pelletier *et al.* , 2021 ; Zollo and Winter, 2002 ); and (4) Vendor influence ( Palvia *et al.* , 2015 ). Using these categories as a starting point, through our hybrid deductive and inductive coding approach we finally identified four categories of capability evolution drivers in our case studies. These categories include (1) Stakeholder initiative; (2) Driving events; (3) Accumulating experience and (4) Emerging issues (see Table 5 ).

The first category entails instances where specific stakeholders take the initiative to found or evolve the COISA capability because they feel motivated to do so. For example, this was the case in Hospital A, where digital advisors and an external consultant took the explicit initiative to set up end-user groups as a foundation of the operational and orchestrational alignment competencies (project lead B; project lead D, hospital A). The second category, i.e. driving events, considers specific events that form a concrete reason to set up, evolve or retrench alignment competencies. A specific example includes a merger of two hospitals (visible in case hospitals A and B), causing strategy- and process harmonization to become an immediate issue (ICT manager, hospital A; CNIO, hospital B). In Hospital C, these driving events include an initially failed EMR implementation and the end-of-life of their old EMR (project lead 1, hospital C). The third category, i.e. Accumulating experience, considers evolutionary paths of the COISA capability caused by lessons learned through accumulated experience over time. For example, in hospital B, the orchestrational alignment competency developed through experience because people became more aware of the interdependencies between their departments (ICT architect, hospital B). The fourth category is emerging issues. This entails specific issues that emerge during the execution and usage of the capability requiring immediate action, then causing the capability to evolve in a certain direction. For example, in Hospital A, several high-priority issues emerged right after go-live, causing the hospital to set up a specific decision-making structure tailored for quick, high-priority decisions, thereby developing the strategic alignment competency (head EMR operations; digital doctor, hospital A).

A few things stand out in our results. First, we see that stakeholder initiative is a driver that is seen in all lifecycle stages considering a forward evolution of the capability. Only in the retrenchment stage, which entails a *decline* of the capability, there are no stakeholder initiative instances as a key driver. Second, out of the lifecycle stages where stakeholder initiative does seem to be a key driver, this seems most so the case for founding- and development stages. Furthermore, driving events seem to be a driver for lifecycle stages where the capability takes an entirely new direction, i.e. at its foundation, at its retrenchment and when part of the capability is renewed. Lastly, the abovementioned findings seem to be valid for all three alignment competencies, and no clear differences or patterns seem to be present, looking at the alignment competencies individually.

## 5. Discussion and conclusion

Our study demonstrates that each of our case hospitals has a unique evolutionary path regarding its COISA capability in pursuit of EMR alignment. Previous work suggests that building such a COISA capability indeed promotes creating a common interpretation and implementation of what it means to apply EMR in an appropriate and timely way across stakeholders, i.e. EMR alignment ( Keshavee *et al.* , 2006 ; Luftman, 2004 ; Luftman and Brier, 1999 ; Walraven *et al.* , 2020 ). Even so, attention should still be paid to the fact that to effectively pursue this endeavor, continuous time and effort should be spent to maintain an adequate level of EMR alignment due to the complex and continuously changing environment that hospitals face ( Keshavee *et al.* , 2006 ; Palvia *et al.* , 2015 ; Walraven *et al.* , 2019 ). Our theoretical contributions are threefold: First, we add to the existing works on EMR in hospital settings. We do so, by taking on the challenge of EMR alignment from a theoretical perspective that is new to this particular area of expertise. Combining insights into which capabilities are essential in EMR alignment and the key drivers of shaping and steering these capabilities provides health researchers with concrete concepts to do rigorous empirical research in health IT. Moreover, it provides a basis of scientific conversation to compare the value of the COISA capability for EMR specifically to its value in relation to other health and non-health IT solutions.

Secondly, we identified a new capability lifecycle stage in our current study, i.e. the coordination stage, meaning that a capability founded within organizational boundaries is brought to a higher network- or ecosystem-level, formally incorporating other organizations in the capability. With this particular addition, we expand on the theoretical developments in capability evolution, especially in terms of possible lifecycle stages that a capability may evolve towards ( Helfat and Peteraf, 2003 ). Furthermore, this finding underlines the importance of the healthcare ecosystem and shows the importance of internal alignment capabilities to enable further upscaling of health information technology (HIT) innovations.

Third, we add a new perspective to viewing alignment as an organizational capability. Specifically, we unfold the different alignment competencies that comprise this particular capability. In addition, we incorporate not only strategic alignment challenges, like in earlier works ( van de Wetering and Mikalef, 2017 ), but also operational and orchestrational competencies. We do so by providing a rich empirical, longitudinal perspective on how this particular viewpoint on alignment resonates with practical findings. A particularly interesting addition to the knowledge base considers our findings in terms of key drivers of alignment: A key finding in this current study is namely the importance of stakeholders as drivers of the COISA capability evolution, also in the long run. Namely, for all capability lifecycle stages except for the retrenchment stage, stakeholder initiative shows to be an essential driver of COISA capability evolution. This resonates with earlier findings on efficacious COISA in a healthcare setting, as described by Walraven *et al.* (2020) . Furthermore, most (but not all) of the stakeholders that drove the forward evolution of the COISA capability in our case studies were middle managers and thus these findings confirm this particular aspect of resource orchestration theory, i.e. that managers can shape the successful orchestration of organizational capabilities ( Chadwick *et al.* , 2015 ; Sirmon *et al.* , 2011 ).

Moreover, both driving events and emerging issues can be related to alignment motivation. Specifically, these should be seen as external factors that motivate stakeholders to engage in alignment competencies. Our findings also resonate with earlier work by Zollo and Winter (2002) , who argued that the key drivers of capability evolution consist of threats and opportunities to the capability. Specifically, intrinsically or extrinsically motivated stakeholders show to provide opportunities for the COISA capability to evolve into different directions, while specific driving events and emerging issues can also form direct threats to the COISA capability, eventually leading to the capability's retrenchment.

### 5.1 Practical implications

Practitioners can use our findings to better build and shape their organization's capabilities to align EMR. A particularly interesting finding is the coordination of alignment competencies to an inter-organizational level. This coordination potentially enables healthcare managers to leverage the alignment competencies within organizational boundaries to work towards cross-organizational alignment on an ecosystem level. However, the specific alignment competencies that are coordinated to this ecosystem level all have their advantages and disadvantages: For example, an advantage of choosing to coordinate the operational alignment competency, as seen in case Hospital B, is that especially among healthcare employees, less resources are needed to maintain operational alignment. Since these resources are already scarce in primary healthcare processes, this clearly is an advantage. Furthermore, if coordination occurs at the operational level, orchestrational issues such as cross-organizational data integration become less challenging because they can be executed centrally through the decisions in the coordinated operational alignment competency. This is less so the case when just the orchestrational and strategic alignment competencies are coordinated and operational configurations are left to individual hospitals. However, choosing to coordinate the operational alignment competency also causes healthcare employees to feel less ownership of the EMR. Furthermore, fundamental changes to the EMR are generally difficult and slow to implement because all hospitals using the EMR have to agree to a specific change before it can actually be implemented.

For all of our case hospitals and in particular hospitals A and B, the EMR implementation provided a substantial opportunity to found and develop an internal COISA capability, which seems to be a sound basis to enable coordination and collaboration on an ecosystem level. Hospitals aiming to coordinate their alignment capabilities may consider to first found and develop such a capability in-house. Furthermore, hospitals could pay specific attention involving suitable stakeholders in building and evolving their COISA capability: Specifically, stakeholders' initiative shows to be an important driver of the evolution of the COISA capability. Furthermore, practitioners could be more conscious of potential external motivators for stakeholders to drive COISA capability evolution. Specifically, driving events and emerging issues could be leveraged to motivate stakeholders toward a co-evolutionary alignment dialogue, eventually leading to better aligned HIT solutions.

### 5.2 Limitations and conclusion

Although we view this particular study's added value for theory and practice to be substantial, our study is not without limitations. First, we only studied three hospitals, all situated in Western Europe. Especially given the seeming importance of stakeholders and thus human factors, it would be interesting to see whether our findings hold in different cultural contexts. Furthermore, our study was based mainly on retrospective interviews, which may have influenced our findings ( Yin, 2018 ). Future research could apply ethnographic approaches and include observations as a research method to get an even deeper insight into how alignment capabilities evolve around EMR in a hospital context. Lastly, we did not get a comprehensive overview of all stakeholder perspectives in all hospitals. For example, in Hospital B, we mostly interviewed people in advisory or IT-roles and only a limited amount of healthcare employees, since these were relatively difficult to access in this particular hospital.

Concluding, our study reveals the different ways in which the EMR-related COISA capability evolved in three hospitals which all recently implemented a new EMR. In doing so, we reveal a new lifecycle stage that shows how an internal COISA capability is scaled up to multiple organizations working with the same EMR vendor. This adds to multiple existing theoretical perspectives, including EMR alignment and capability evolution. Furthermore, we underline the importance of stakeholders in the COISA capability's evolution. Practitioners can use our findings to effectively improve their EMR alignment through effective COISA coordination and stakeholder involvement.

## Figures and Tables

**Table 1:** Definitions of co-evolutionary alignment competencies

Competency	Definition
Strategic alignment competency	*An organizations' ability to formulate strategic goal, and articulate strategic plans and structures to implement these goals in relation to IS, while monitoring relevance and topicality of these plans, goals and structures, in line with frequencies of internal and external changes.* ( Baker *et al.* , 2011 ; Liang *et al.* , 2017 ; Sabherwal and Chan, 2001 ; Tanriverdi *et al.* , 2010 ; Walraven *et al.* , 2021 ; Yeow *et al.* , 2018 )
Orchestrational alignment competency	*An organization's ability to maintain the coherence between their information systems, goals, processes, data, infrastructure, roles and functions, through architectural practices such as the definition and application of architectural principles or standards, while monitoring relevance and topicality of these architectural practices, in line with frequencies of strategic and operational changes* ( Rolland *et al.* , 2015 ; Schilling *et al.* , 2017 ; Vessey and Ward, 2013 ; Walraven *et al.* , 2021 ; Weeger and Ulrich, 2016 )
Operational alignment competency	*An organization's ability to collaboratively use IT solutions effectively in daily operations and implement and optimize IT solutions in operational settings in line with end-users* ' *needs, while monitoring and leveraging improvement possibilities during IT usage, implementations and operations.* ( Allen *et al.* , 2013 ; Amarilli *et al.* , 2017 ; Burton-Jones and Gallivan, 2007 ; Goh *et al.* , 2011 ; Lyytinen and Newman, 2008 ; Vidgen and Wang, 2009 ; Walraven *et al.* , 2021 )

**Table 2:** Capability lifecycle stages, based on Helfat and Peteraf (2003)

Capability Lifecycle stage	Characteristics
Founding	A group with leadership and with the ability to take collaborative action is formed, with a common goal to create a new capability within the organization
Development	The capability building group or team makes decisions on how to best shape the capability at hand, informed by accumulating experiences in doing so
Maturity	The capability no longer changes, but is maintained by regular exercise by the organization
Retirement	The capability ceases to exist
Retrenchment	The usage of the capability declines over time
Redeployment	The capability is transferred to another product market
Renewal	The capability returns to the development stage after having left this stage at an earlier point in time
Replication	The capability is transferred to another geographic market (but applied to the same product or service)
Recombination	The capability is combined with other capabilities to serve a different but related market

**Table 3:** Case hospital characteristics

	Hospital A	Hospital B	Hospital C
Size	750–1,000 beds	500–750 beds	>1,000 beds
EMR Vendor	Vendor 1	Vendor 2	Vendor 1
Simultaneous merger?	Yes	Yes	No
Scope EMR	Hospital-wide	Hospital-wide	Hospital-wide
Pre-implementation phase	Extensive (separate program)	Limited	Very limited
Implementation program approach	One go-live	One go-live	Two-stage approach
Hospital type	Top clinical	Top clinical	Academic

**Table 4:** Interviewees for each case hospital and phase

Hospital	A		B		C	
Phase Group	I	II	I	II	I	II
IT	-ICT manager -Project lead A	-Head EMR ops -Project lead A -Project leader training	-ICT architect	-CIO -ICT architect -Head EMR ops	-Project lead 1 -Project lead 2	-Program manager 2 -Program manager 3
External	-Project lead B	N/A	-Program manager -Vendor rep	N/A	-Program manager 1 -Project lead 3 -Project lead 4	N/A
Mgmt	-Project lead C	-Manager healthcare	-Project lead	-Information manager	-Project lead 5	-Project lead 6
Medical	-Project lead D	-Project lead D -Digital Doctor -Digital Nurse	-CNIO	-CNIO	-Digital doctor 1	-CMIO -Digital doctor 1 -Digital doctor 2

**Table 5:** Definitions of key drivers of the COISA capability evolution

Driver	Definition
Stakeholder initiative	One or more stakeholder(s) (group(s)) take(s) initiative to drive capability evolution in a certain direction
Driving events	A specific event in time causes a capability to evolve in a certain direction
Accumulating experiences	Through accumulating experience with a capability and thanks to the resulting knowledge, the capability evolves in a certain direction
Emerging issues	Specific issues emerge during the execution and usage of the capability evolution that require immediate action, causing the capability to evolve in a certain direction
